# Soil Type and Cyanobacteria Species Influence the Macromolecular and Chemical Characteristics of the Polysaccharidic Matrix in Induced Biocrusts

**DOI:** 10.1007/s00248-018-1305-y

**Published:** 2018-12-08

**Authors:** Sonia Chamizo, Alessandra Adessi, Gianmarco Mugnai, Andrea Simiani, Roberto De Philippis

**Affiliations:** Department of Agrifood Production and Environmental Sciences (DISPAA), University of Florence, Florence, Italy

**Keywords:** Biological soil crust, Cyanobacteria inoculation, Molecular weight, Monosaccharide composition, Loosely bound EPS, Tightly bound EPS

## Abstract

Inoculation of soils with cyanobacteria is proposed as a sustainable biotechnological technique for restoration of degraded areas in drylands due to the important role that cyanobacteria and their exopolysaccharides (EPS) play in the environment. So far, few studies have analyzed the macromolecular and chemical characteristics of the polysaccharidic matrix in induced cyanobacterial biocrusts and the scarce existing studies have mainly focused on sandy soil textures. However, the characteristics of the cyanobacterial polysaccharidic matrix may greatly depend on soil type. The objective of this study was to examine the macromolecular distribution and monosaccharidic composition of the polysaccharidic matrix induced by inoculation of two cyanobacterial species common in arid environments, *Phormidium ambiguum* (non N-fixing) and *Scytonema javanicum* (N-fixing) in different soil types. *S. javanicum* promoted a higher release in the soil of the more soluble and less condensed EPS fraction (i.e., the loosely bound EPS fraction, LB-EPS), while *P. ambiguum* showed a higher release of the less soluble and more condensed EPS fraction (i.e., the tightly bound EPS fraction, TB-EPS). LB-EPSs were mainly composed of low MW molecules (< 50 kDa), while TB-EPSs were mainly composed of high MW molecules (1100–2000 kDa). The two EPS fractions showed a complex monosaccharidic composition (from 11 to 12 different types of monosaccharides), with glucose as the most abundant monosaccharide, in particular in the poorer soils characterized by lower organic C contents. In more C-rich soils, high abundances of galactose, mannose, and xylose were also found. Low abundance of uronic acids and hydrophobic monosaccharides, such as fucose and rhamnose, was found in the EPS extracted from the inoculated soils. Our results point to the influence of soil type on the macromolecular distribution and monosaccharide composition of the polysaccharidic matrix in induced biocrusts, which is likely to affect biocrust development and their role in soil structure and nutrient cycling in restored dryland soils.

**Electronic supplementary material:**

The online version of this article (10.1007/s00248-018-1305-y) contains supplementary material, which is available to authorized users.

## Introduction

Cyanobacteria are the oldest oxygenic phototrophic microorganisms found in the Earth and the primary colonizers of terrestrial ecosystems [1]. They play a particularly important role as primary producers in desert areas where they act as pioneer organisms forming biological soil crust (or biocrust) communities [2]. In these environments, cyanobacteria improve soil functions by increasing carbon and nitrogen contents, soil aggregation and stability and soil water status [3, 4]. Many of these functions are related to their ability to secrete large amounts of exopolysaccharides (EPS) [5–8].

Cyanobacterial EPSs are key in the consolidation of soil particles and the setting of initial conditions for establishment of biocrust communities [5]. Bare soils are initially colonized by large mobile filamentous cyanobacteria, usually belonging to the genus *Microcoleus,* which can be able to migrate to the surface upon wetting and retreat immediately below upon drying [9]. This movement spreads sheath and EPS material throughout the uppermost soil layer, improving soil aggregation in the top soil profile [10]. Then, smaller, less mobile cyanobacteria colonize the soil [11]. In later successional stages, if conditions permit, lichens and mosses colonize the soil [12]. Concomitant to biocrust development, cyanobacteria and the EPS matrix they produce play a number of key roles in drylands: (i) cyanobacteria fix CO_2_ and increase the organic carbon content of soils, both through EPS release and cyanobacterial biomass decomposition, thus representing a major source of C in dryland soils where this resource is mainly concentrated beneath plants [13]; (ii) some cyanobacteria fix N_2_ and release it into the surrounding environment, making it available for the soil heterotrophic community and plants [14]; (iii) the EPS matrix acts as a repository for nutrients, sequestering metabolites released by microbes, in particular amino acids and organic acids [15], and metal cations such as Ca^2+^ and Mg^2+^ [16, 17], greatly enhancing the nutrient content of soils; (iv) cyanobacterial polysaccharidic sheaths absorb large amounts of water and significantly increase water uptake and retention in soils [18, 19]; (v) both the network formed by cyanobacteria filaments and their sticky EPS glue soil particles together and increase the number and size of aggregates, improving soil structure and stability [20–23] and reducing the soil susceptibility to wind and water erosion [7, 24, 25]; (vi) due to the improvement in soil properties and the release of nutrients required by plants, cyanobacteria improve seed germination and plant growth and facilitates the succession of biocrusts and plant communities [26–28].

Macromolecular and chemical characterization of EPSs has been thoroughly studied in batch cultures in the laboratory for isolated cyanobacterial species [29, 30]. However, characterization of the polysaccharidic matrix in natural or artificially induced cyanobacterial soil crusts remains much less explored. Two types of EPS fractions can be identified in the soil polysaccharidic matrix promoted by cyanobacteria: a less condensed fraction, easily released into the environment, referred to as “loosely bound EPS” (LB-EPS), and a more condensed fraction, firmly attached to the cells and soil particles, referred to as “tightly bound EPS” (TB-EPS) [8, 17]. These two fractions may confer contrasting properties to soils depending on their macromolecular and chemical features, directly affecting the role that cyanobacteria play in hydrological and biogeochemical processes in dryland soils and their interactions with soil microbial and plant communities.

Previous studies have shown the effectiveness of soil inoculation with EPS-producing cyanobacteria in soil aggregation and fertility, and water relations [23, 31–34]. Most studies have used Oscillatoriales species, mainly *Microcoleus vaginatus,* or a mixture of Oscillatoriales and Nostocales species, with *M. vaginatus* in higher proportions, for stabilization of desert sand dunes in China [28, 32, 35, 36]. However, the effect of induced biocrusts on soil properties largely depends on the selected cyanobacterial strain and inherent properties of the inoculated soil [23, 37]. In spite of the importance of the polysaccharidic matrix for the successful performance and development of biocrusts and their effects on soil properties, only few studies have examined the macromolecular and chemical features of cyanobacterial EPS in induced biocrusts. These studies have been conducted either in laboratory [34, 38] or field conditions [26, 39, 40], and all of them have focused uniquely on sandy substrates. To our knowledge, no previous studies have analyzed the characteristics of the EPS matrix in induced cyanobacterial crusts in different soil types with contrasting physical and chemical properties. Knowledge of how the characteristics of the polysaccharidic matrix are affected by different soil types is crucial to understand the role of EPSs in biocrusts [8], and also to select the most suitable strains according to the characteristics of the soils that need to be restored.

The aim of this study was to investigate the macromolecular and chemical features of the soil polysaccharidic matrix induced by inoculation of two different cyanobacteria with different physiological and morphological traits on four semiarid soil types characterized by different soil texture (from fine to coarse particle size distribution) and organic carbon and nitrogen contents. The specific objectives of this study were (i) to explore whether the amount of the two EPS fractions (LB-EPSs and TB-EPSs) in the soil depended on the inoculated cyanobacteria strain and soil type; and (ii) to analyze the effect of cyanobacteria strain and soil type on the macromolecular distribution and the monosaccharidic composition of the two EPS fractions, LB-EPS and TB-EPS.

## Material and Methods

### Soil Inoculation Experiments

**Fig. 1:**
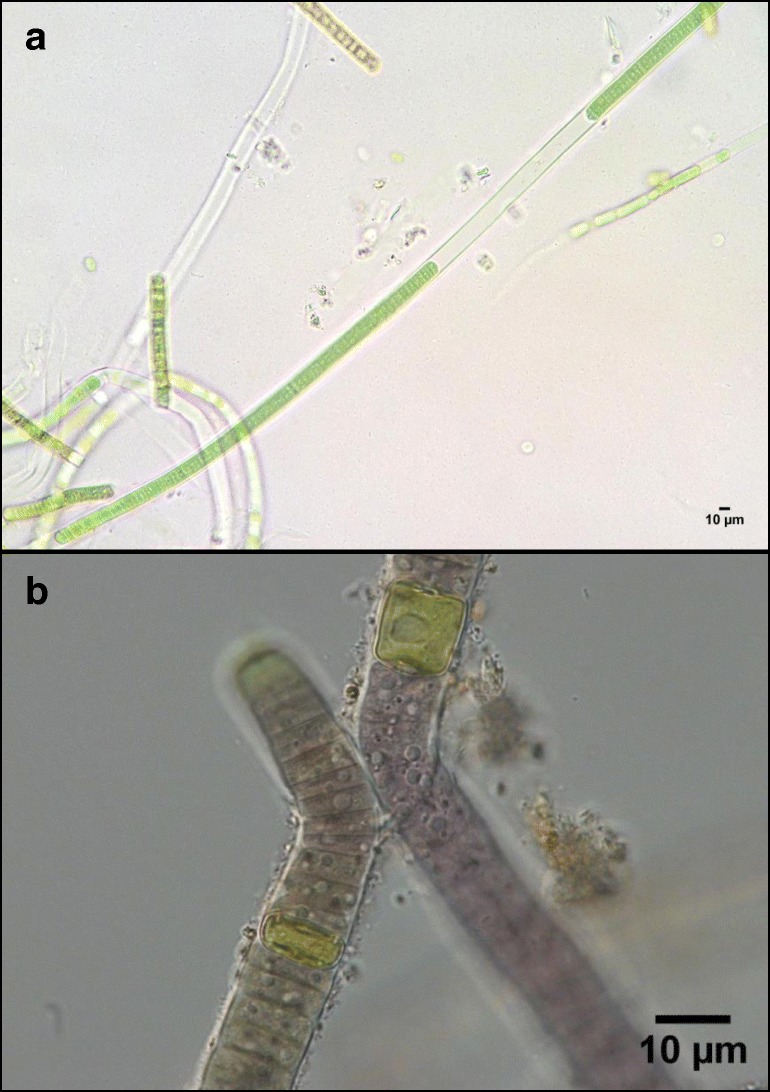
Optical microscope images of the two selected strains: *Phormidium ambiguum* NIES-2121 (**a**) and *Scytonema javanicum* NIES-1956 (**b**)

Two filamentous cyanobacterial strains were selected for inoculation on different soil types: *Phormidium ambiguum* Gomont NIES-2121, belonging to the Order Oscillatoriales, and *Scytonema javanicum* Bornet & Flahault NIES-1956, belonging to the Order Nostocales. *P. ambiguum* was originally isolated from an African soil, while *S. javanicum* was originally isolated from the Tsukuba Botanical Garden (Japan). The selection of the two cyanobacterial species was based on the wide distribution of *Phormidium* sp. and *Scytonema* sp. in hot and cold deserts [41] as well as on their very contrasting morphology and physiological traits. *P. ambiguum* filaments are thinner and lack heterocysts, while *S. javanicum* filaments are thicker and show the presence of heterocysts (Fig. 1). In addition, the two cyanobacteria occupy different niches in the soil. The genus *Scytonema* usually lives on the soil surface, while *Phormidium* sp. lives immediately below [6], being especially abundant at 0–1 cm soil depth [42, 43]. The two cyanobacterial strains were grown in BG11_0_ (*S. javanicum*) or BG11 (*P. ambiguum*) media in a rotatory shaker (Innova 44B, New Brunswick, USA) at a constant temperature of 30 °C, light intensity of 15 μmol photons m^−2^ s^−1^, and with a stirring speed of 100 rpm. Each strain was individually inoculated on four soil types, collected from two semiarid areas in SE Spain (Tabernas Desert and Cabo de Gata-Níjar Natural Park) and characterized by different soil texture, organic carbon (OC) and total nitrogen contents. Soils were collected from a depth of 0–10 cm and were classified as (1) silt loam (27% sand, 59% silt, 14% clay), having an average OC content of 0.5% and nitrogen content of 0.89 g kg^−1^; (2) sandy loam (64% sand, 21% silt, 15% clay), having an average OC content of 1.3% and nitrogen content of 1.13 g kg^−1^; (3) loamy sand (73% sand, 16% silt, 11% clay), having an average OC content of 0.6% and nitrogen content of 0.81 g kg^−1^; and (4) sandy (92% sand, 1% silt, 7% clay), having an average OC content of 0.1% and nitrogen content of 0.21 g kg^−1^. Small Petri dishes (12 mm height × 54 mm diameter) were filled with 30 g of each sterilized soil type. Soils were sterilized with the objective of isolating the effect of the selected cyanobacteria strains on the characteristics of the EPS matrix and minimizing the influence on the EPS properties of other autotrophs naturally present in the soil. For the sterilization, soils were autoclaved twice for 20 min at 120 °C. Then, three treatments were set up for each soil type: soil inoculation with *P. ambiguum*, soil inoculation with *S. javanicum*, and control soil (without inoculum). Three replicates were considered for each treatment. Inoculation was done by adding 30 mg (dry weight) of cyanobacterial biomass on each Petri dish (equivalent to 5 g m^−2^). Samples were incubated in a plexiglass growth chamber with controlled temperature (30 °C), light intensity (45 μmol photons m^−2^ s^−1^) and relative humidity (0%) for 90 days. Twice a week, samples were irrigated with 2 mm of distilled water, corresponding to the average annual rainfall of the study areas where the soils were collected (~ 215 mm) and calculated according to the duration of the experiment (90 days). More information about the soil inoculation experiments can be found in Chamizo et al. [23].

### Crust Sampling

The top crust (~ 8 mm thick in the silt loam, sandy loam, and loamy sand soils, and ~ 2 mm in the sandy soils) was collected from the different Petri dishes after 90 days of incubation. In order to compare the sandy soil with the other soil types, a weighed mean with depth (considering the 0–2 mm crust and the 2–8 mm soil layer) was calculated in the sandy soil for the measured variables. The crust was manually ground to a fine powder with mortar and pestle for the soil chemical analyses.

### Chlorophyll a Determination

Chlorophyll *a* was extracted using ethanol as solvent. One gram of soil was weighted into a vial with 5 mL of ethanol, then vortexed and heated at 80 °C for 5 min. Samples were cooled at 4 °C for 24 h and centrifuged to separate the supernatant for analysis of the absorbance at 665 nm by spectrophotometer [44]. Chlorophyll *a* content was calculated according to the following equation:

1 $$ \mathrm{Chlorophyll}\ a=\left(11.9035\times \mathrm{A}{665}_0\times \mathrm{V}\right)\ \mathrm{x}\ \left(\mathrm{g}\ {\mathrm{soil}}^{-1}\right)\ \mathrm{x}\ \mathrm{L} $$

where V is the volume of solvent (mL) and L is the path length [45].

### Exopolysaccharide Extraction and Quantification

The two EPS fractions (LB-EPSs and TB-EPSs) were extracted from the soil samples and their carbohydrate content quantified. LB-EPSs were extracted with distilled water at room temperature for 20 min. The supernatant was recovered after centrifugation at 3500×*g* for 30 min. This process was repeated three times for each sample and the three supernatants obtained were collected together. TB-EPSs were recovered from the resulting pellet using three extractions with 0.1 M Na_2_EDTA and centrifugation at 3500×*g* for 30 min [38]. The three supernatants obtained after the three extractions were collected together. The carbohydrate content of both LB-EPS and TB-EPS extracts was determined using the phenol-sulfuric acid assay [46]. Three instrumental replicates of each sample were conducted.

### Molecular Weight

Apparent molecular weight (MW) distribution of both EPS fractions was determined by size exclusion chromatography (SEC). Previous to determination of MW distribution in the TB-EPS fraction, the excess of Na_2_EDTA that could interfere with the chromatographic analysis was removed by dialyzing the extracts in nitrocellulose tubular membranes (14 kDa MW cutoff, Medicell International, UK) for 24 h against distilled water, with two changes of water, before being dried. Dried extracts were dissolved in deionized water, transferred to Eppendorf tubes, and clarified by ultracentrifugation at 13,000×*g* in order to remove the coarse particulate. The extracts were then analyzed by injection in a Varian Pro-Star liquid chromatograph (Varian Inc., USA) equipped with a refractive index (RI) detector and two columns, PolySep-GFC-P 6000 and 4000 (Phenomenex, USA), connected in series. The columns (700 mm length and 7.8 mm internal diameter) had separation ranges of 100 kDa to 15 MDa and 0.3 to 400 kDa, respectively. Samples were analyzed with runs of 70 min using HPLC-grade water as eluent at a flow rate of 0.4 mL min^−1^. Dextran (Sigma-Aldrich, USA) at known MWs (2000, 1100, 410, 150, 50 kDa) was used as standards.

### Monosaccharide Composition

The monosaccharide composition of LB-EPSs and TB-EPSs was determined by ion exchange chromatography (IEC). Before IEC analysis, the extracts were hydrolyzed by adding 1 mL of extract to 1 mL of 4 N trifluoroacetic acid (TFA) in screw-cap vials, for 120 min at 120 °C. Afterward, the excess of TFA was removed by drying on a rotary evaporator and the dried extracts re-solubilized in deionized water. This operation was repeated three times for each sample. Then, the extracts were analyzed by using a Dionex ICS-2500 ion exchange chromatograph (Dionex, USA) equipped with an ED50 pulsed amperometric detector operating with a gold working electrode (Dionex) and a CarboPac PA1 column of 250-mm length and 4.6-mm internal diameter (Dionex). Eluents used were HPLC-grade water (A), 0.185 M Na hydroxide (B), and 0.488 M Na acetate (C). In the first stage of the analysis (from injection time to 20 min), the eluent consisted of 90% A and 10% B; in the second stage (from 20 to 30 min), the eluent consisted of 50% B and 50% C; in the final stage (from 30 to 60 min), the eluent was that of the first stage. The flow rate was kept at 1 mL min^−1^. Peaks for each sugar were identified on the basis of the retention time of known standards.

### Data Analysis

The ratio of inoculated to control soil was determined for chlorophyll *a*, LB-EPS, and TB-EPS contents in order to assess the magnitude of change of these variables in the inoculated with respect to the non-inoculated soils. A ratio equal to 1 indicates similar values in control and inoculated soils, while a ratio greater than 1 indicates higher content in the inoculated than in the non-inoculated soils.

The effect of soil type and inoculum type on chlorophyll *a*, LB-EPS, and TB-EPS contents and MW and monosaccharide abundance was analyzed with two-way ANOVA and the Fisher’s LSD test at the 5% significance level. A principal component analysis (PCA) was performed on monosaccharidic composition data in order to compress the information on to a smaller number of non-colinear variables or principal components (PCs). In PCA, the scores are calculated such that the first PC accounts for the largest variation in the data and has the maximum variance of the scores, and the following PCs explain as much of the remaining variation as possible. Data were plotted to observe possible differences between soil or inoculum types according to monosaccharidic composition. Statistical analyses were carried out using the STATISTICA software, version 8.0 (StatSoft, Inc., Tulsa, OK, USA).

## Results

### Effect of Cyanobacteria Inoculation on Chlorophyll a Content and EPS Amount

**Fig. 2:**
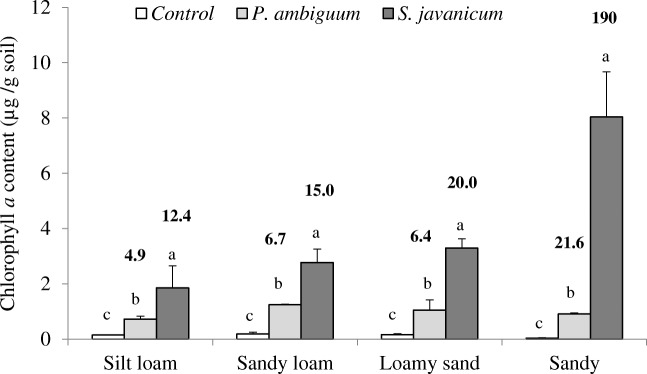
Chlorophyll *a* content (mean ± sd, *n* = 3) after 90 days of incubation. Significant differences (*p* < 0.05) among cyanobacteria treatments for each soil type are indicated with different letters. The number in bold above the bars indicates the ratio inoculated to control soil

Biocrust development following cyanobacteria inoculation was confirmed by the increase in surface darkness and greenish color of the surface and increases in chlorophyll *a* and EPS contents after 90 days of incubation. The two-way ANOVA showed that soil type and inoculum treatment had significant effects on chlorophyll *a*, LB-EPS, and TB-EPS contents (*p* < 0.001). The interaction of both predictor factors was also significant for all variables (*p* < 0.001), indicating that the effect of the inoculum varied with soil type. The inoculated soils showed significantly higher chlorophyll *a* content than control soils and the highest values were found in the sandy soil inoculated with *S. javanicum*. Soils inoculated with this strain showed between 120% (silt loam soil) and 780% (sandy soil) higher content than those inoculated with *P. ambiguum* after 90 days (Fig. 2).

**Fig. 3:**
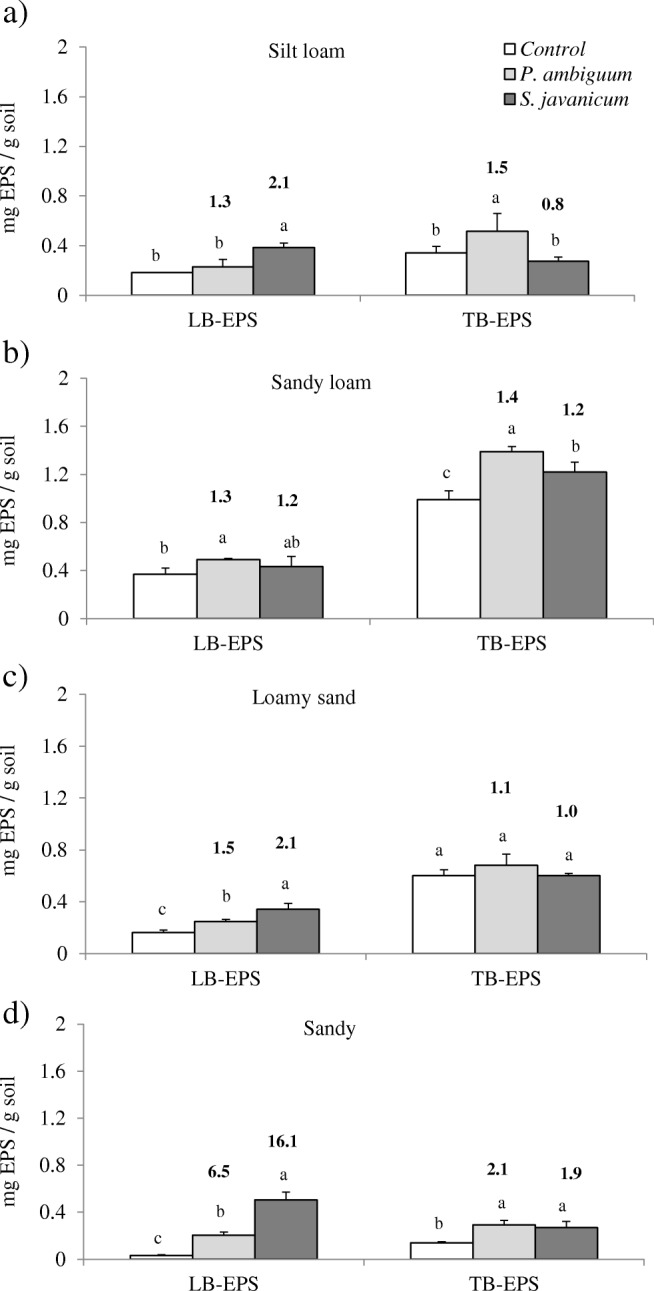
Loosely bound (LB) and tightly bound (TB) EPS content (mean ± sd, *n* = 3) in the control and inoculated soils in the different soil types: silt loam (**a**), sandy loam (**b**), loamy sand (**c**), and sandy (**d**). Significant differences (*p* < 0.05) among cyanobacteria treatments for each EPS fraction are indicated with different letters. The number in bold above the bars indicates the ratio inoculated to control soil

The amount of the two EPS fractions also varied with soil type and inoculum treatment. The sandy loam soil showed the highest LB-EPS and TB-EPS contents, while the sandy soil showed the lowest contents, having the silt loam and the loamy sand soils intermediate values (Fig. 3). TB-EPSs represented a predominant fraction of total EPS in the inoculated sandy loam and loamy sand soils (74 ± 3% and 69 ± 6%, respectively), while represented a lower faction of total EPS in silt loam and sandy soils (55 ± 17%, and 47 ± 14%, respectively). The sandy soil showed the highest increase in both LB-EPSs and TB-EPSs following cyanobacteria inoculation in comparison with non-inoculated soils. Differences were observed in the amount of the two EPS fractions depending on the inoculated strain. *S. javanicum* promoted significantly higher LB-EPSs than *P. ambiguum* in all soils except for the sandy loam, where both showed similar LB-EPS amount. In contrast, *P. ambiguum* promoted higher TB-EPSs than *S. javanicum*, although differences were only significant in silt loam and sandy loam soils (Fig. 3). For all soils, the ratio inoculated to control soil for LB-EPSs was higher in *S. javanicum*—compared to *P. ambiguum-*inoculated soils, with the exception of the sandy loam soil, while the opposite was found for TB-EPSs (Fig. 3).

### Macromolecular Distribution of the Polysaccharidic Matrix in the Induced Biocrust

**Fig. 4:**
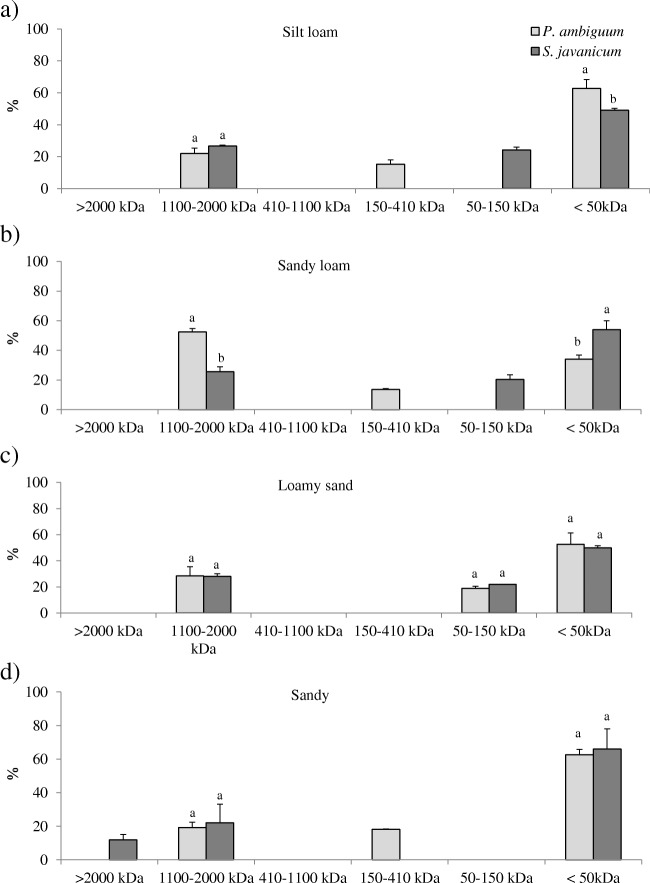
Size distribution (%) of LB-EPSs in the inoculated soils for the different soil types. Values are the average of three replicates and bars represent standard deviation. Different letters indicate significant differences (*p* < 0.05) between the two strains within each MW class

Both EPS fractions showed significant differences in the apparent MW distribution. The LB-EPSs were mainly composed of low MW molecules (< 50 kDa) that represented around 50% of total LB-EPSs, and a smaller percentage of high MW molecules with a size between 1100 and 2000 kDa that represented around 30% of total LB-EPSs (Fig. 4). The percentage of low MW molecules was similar in the soils inoculated with *P. ambiguum* and *S. javanicum* in the coarser soil types (loamy sand and sandy loam). In the silt loam soil, inoculation with *P. ambiguum* led to LB-EPS with a higher percentage of low MW molecules (< 50 kDa), while the opposite was found in the sandy loam soil, where *S. javanicum* led to a higher percentage of low MW molecules (Fig. 4).

**Fig. 5:**
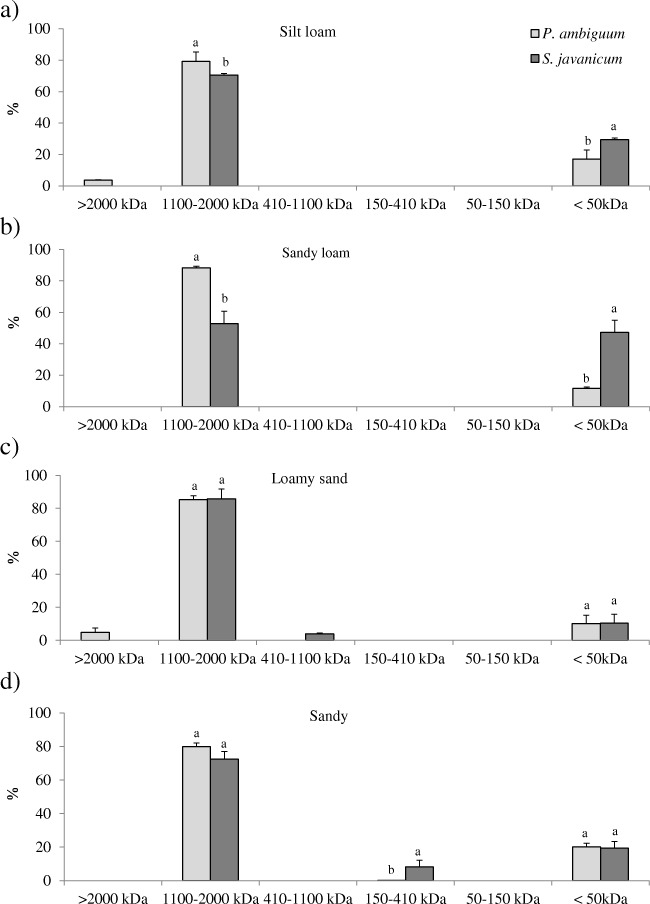
Size distribution (%) of TB-EPSs in the inoculated soils for the different soil types. Values correspond to the average of three replicates and bars represent standard deviation. Different letters indicate significant differences (*p* < 0.05) between the two strains within each MW class

Contrary to the pattern observed in MW distribution for the LB-EPS fraction, the TB-EPSs were mainly composed of high MW molecules (between 1100 and 2000 kDa) (Fig. 5). This fraction represented up to 88% of total TB-EPSs in the inoculated soils (on average, 77%). The percentage of high MW molecules in TB-EPS was similar in the soils inoculated with the two cyanobacteria, except for the sandy loam soil, where the TB-EPSs produced by *P. ambiguum* showed a higher percentage of high MW molecules compared to *S. javanicum* (Fig. 5).

### Monosaccharidic Composition of the Polysaccharidic Matrix in the Induced Biocrust

**Fig. 6:**
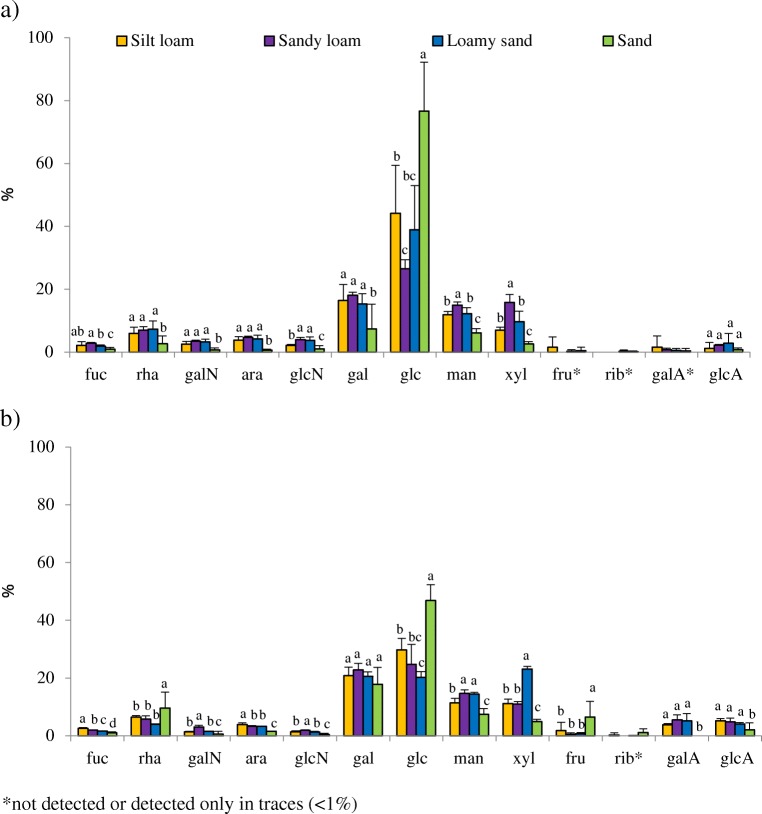
Relative abundances of the single monosaccharides, expressed as mol of the single monosaccharide divided by the total amount of moles of monosaccharides in the EPS × 100, in the LB-EPS (**a**) and TB-EPS (**b**) fractions extracted from the inoculated soils. Values correspond to the average of six replicates (3 samples of *P. ambiguum* and 3 samples of *S. javanicum*) and bars represent standard deviation. Different letters indicate significant differences (*p* < 0.05) in monosaccharide content among the four soil types. *not detected or detected only in traces (< 1%)

IEC analyses displayed the complexity of the polysaccharidic matrix of the two EPS fractions in terms of monosaccharidic composition. LB-EPSs were generally composed of ten monosaccharides. Fructose, ribose, and galacturonic acid were not present or detected only in traces (Fig. 6a). TB-EPSs were generally composed of 12 monosaccharides, with ribose absent or detected only in traces (Fig. 6b). Glucose, galactose, mannose, and xylose were the monosaccharides present at the highest percentage in both fractions. Glucose was the dominant monosaccharide, representing a higher percentage of total monosaccharides in LB-EPSs (47 ± 21%) than in TB-EPSs (30 ± 12%) (Fig. 6). Cyanobacteria inoculation induced differences in the molar percentage of monosaccharides depending on soil type. In the LB-EPS fraction, inoculated sandy soils showed much higher glucose abundance than the other finer textured soils, but these showed higher abundance of galactose, mannose, and xylose (Table S1). The sandy loam soil showed the lowest glucose and the highest mannose and xylose content of all soils. This soil showed also higher amino-sugars content (Fig. 6a). Differences between the two inoculum types in the monosaccharidic composition of the LB-EPSs were found in the silt loam and sandy soils. In the silt loam soil, *P. ambiguum* showed a higher number of monosaccharides (12) than *S. javanicum* (9). In addition, *P. ambiguum* showed higher galactose and less glucose abundance compared to *S. javanicum* (Table S1). In the sandy soil, an opposite trend was found. A lower number of monosaccharides was found in the sandy soil inoculated with *P. ambiguum* (4) than with *S. javanicum* (9) (Table S1). In this soil, *P. ambiguum* promoted higher glucose abundance, while *S. javanicum* promoted higher galactose abundance.

In the TB-EPS fraction, inoculated sandy soils showed higher abundance of glucose and no presence of galacturonic acid compared to the other soil types (Fig. 6b). Main differences between the two inoculum types were found in the sandy soil. *P. ambiguum* promoted a higher abundance of galactose and mannose, while *S. javanicum* promoted a higher abundance of rhamnose and fructose (Table S2).

**Fig. 7:**
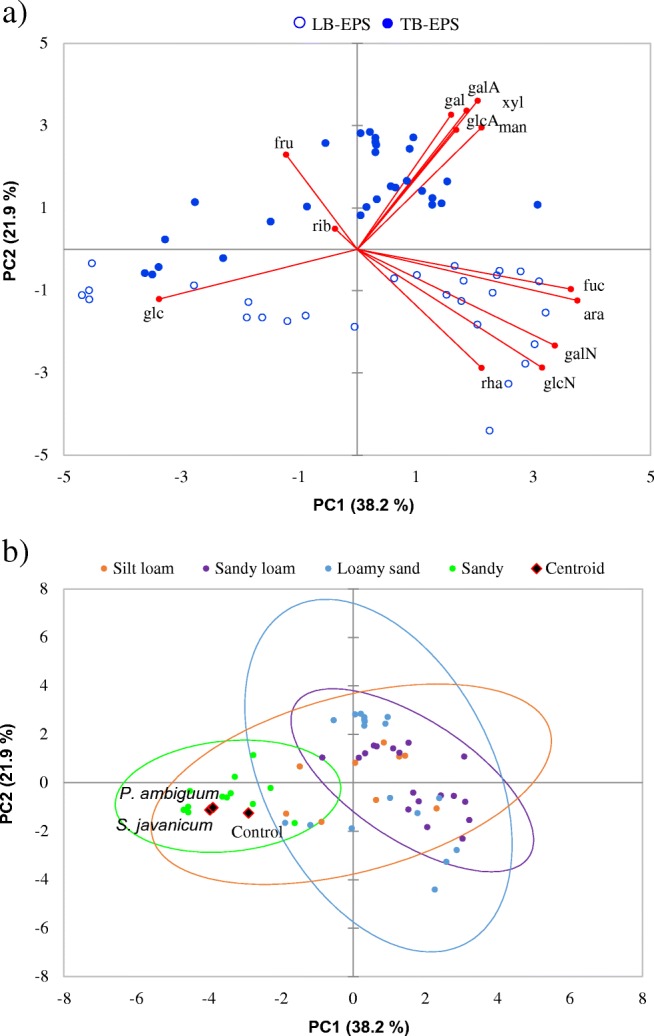
Score plot of PC1 vs PC2 showing the distribution of control and inoculated soils according to the monosaccharidic composition. Monosaccharides in control samples from silt loam and sandy soils were detected in traces and then excluded of the PCA analysis. **a** PCA biplot showing the distribution of LB-EPSs and TB-EPSs. **b** PCA biplot showing the spheroids for each soil type. The centroids for controls and inoculated soils are also shown. fuc fucose, rha rhamnose, galN galactosamine, ara arabinose, glcN glucosamine, gal galactose, glc glucose, man mannose, xyl xylose, fru fructose, rib ribose, galA galacturonic acid, glcA glucuronic acid

Application of PCA to the monosaccharidic composition of LB-EPS and TB-EPS extracted from inoculated soils and from controls (excluding silty loam and sandy soils where all monosaccharides were found in traces) showed that the first and second components explained 41.7% and 24.7% of the variance, respectively (Fig. 7a). The first component positively correlated with fucose, arabinose, galactosamine, and glucosamine, and negatively with glucose. The second component positively correlated with xylose, mannose, galactose, and uronic acids, and negatively with rhamnose, glucosamine, and galactosamine. The second component allowed differentiating the clusters for the two EPS fractions, showing negative effects on LB-EPSs and positive effects on TB-EPSs. No clear separation was found among soil types or inoculum types. However, inoculated sandy soils tended to group separately from the resting soils, associated to glucose abundance. It could also be seen that the centroid for controls was separated from inoculated soils (Fig. 7b).

## Discussion

### Effect of Cyanobacteria Inoculation on Biocrust Growth

Several studies have demonstrated the possibility of accelerating biocrust formation in disturbed bare areas by means of soil inoculation with cyanobacteria or other biocrust organisms [32, 33, 47]. In this study, inoculation of two cyanobacterial strains in four soil types led to biocrust formation, resulting in significant increases in chlorophyll *a* and EPS contents. However, contrasting differences were found depending on soil type and the inoculated strain. Inoculation of *S. javanicum* promoted the highest increase in chlorophyll *a* in all soil types and especially in the sandy soil (Fig. 2). This species has been reported to grow on the surface, allowing it to have a more efficient use of light to fix C and N [6] and favoring its faster growth. *P. ambiguum,* due to its location immediately below the surface [6] and to the lack of heterocysts, is expected to have a slower metabolism, which explains the lower increase in chlorophyll *a* after 90 days.

### Effect of Cyanobacteria Inoculation and Soil Type on EPS Amount and Macromolecular Distribution of the Crust Polysaccharidic Matrix

Cyanobacteria play a crucial role as providers of C sources in nutrient-poor dryland soils, mainly by the secretion of EPSs that can represent up to 75% of total carbohydrate content in the topsoil [13]. In this study, cyanobacteria inoculation led to an increase in EPS content in all tested soils and, more remarkably, in the less organic C enriched-soils, the silt loam and the sandy soils, which inherently had lower EPS contents. As recently reported, the effect of cyanobacteria in increasing C and N content is more remarkable in soils having lower nutrient content, while having a less significant effect in more nutrient-rich soils [23, 37]. Interestingly, the inoculated soils showed significant differences in the amount of the two EPS fractions depending on the inoculated strain. Soils inoculated with *S. javanicum* showed higher LB-EPS content, while soils inoculated with *P. ambiguum* showed higher TB-EPS content (Fig. 3). This could lead to differences in the availability of C sources for soil microbial community depending on the abundance of one of other cyanobacterial species in the biocrusts. The LB-EPS fraction was predominantly (50–60%) composed of low MW molecules (< 50 kDa) and a relatively lower percentage (20–30%) of high MW molecules (1100–2000 kDa) (Fig. 4). Meanwhile, the TB-EPS fraction was mainly composed of high MW molecules (1100–2000 kDa) that represented around 80% of total TB-EPSs (Fig. 5). In cyanobacterial biocrusts, the enzymes that hydrolyze low MW substrates are more active than those that hydrolyze high MW substrates [48]. Consequently, higher synthesis by *S. javanicum* of less condensed and more soluble LB-EPSs, mostly composed of low MW molecules, could mainly play a significant role as source of C for soil microbial activity. Meanwhile, high MW sugars contained in the TB-EPS fraction, whose amount was more significantly enhanced by *P. ambiguum*, would be more resistant to degradation and could mainly play a structural role, contributing to soil particle consolidation. This different role of the two EPS fractions has been discussed in a previous study, which showed a higher percentage of low MW sugars (< 64 kDa) in the LB-EPS fraction associated to mature biocrusts where higher enzymatic activity resulted in low MW carbohydrates, while the TB-EPS fraction, which contained 90% of high MW sugars (760–2000 kDa), were mainly thought to contribute to sand stabilization [39]. This is supported by previous results found by us in which soil inoculation with *P. ambiguum* led to higher surface penetration resistance than inoculation with *S. javanicum* [23]*.* Nevertheless, high MW EPS may also have an important role as C source for microbial activity. Under natural conditions, complex high MW sugars can be metabolized to simple sugars such as glucose, galactose and fructose [5, 49], which can be readily utilized by the crustal microbial community or, alternatively, can be used as protection against desiccation [3].

### Effect of Cyanobacteria Inoculation and Soil Type on the Monosaccharidic Composition of the Polysaccharidic Matrix

The inoculated soil types showed differences in the amount and macromolecular distribution of the two EPS fractions, but also on the relative abundance of the different monosaccharides. The presence of one or two uronic acids, which confer a sticky character to the macromolecules, and the presence of pentoses (xylose, arabinose, and ribose) are peculiar features of cyanobacterial EPS than differentiate them from the EPS produced by other microorganisms [50–52]. In general, the monosaccharide found at the highest concentrations in cyanobacterial EPS is glucose, although xylose, arabinose, galactose, or fucose can be present at higher concentrations than glucose in some polymers [17, 53]. The presence of ten and eight monosaccharides have been described, respectively, in the EPS synthesized by *S. javanicum* and a species of the genus *Phormidium*, *P. tenue*, both strains isolated from algal crusts [54]. Uronic acids were found in trace concentrations. In a more recent work, Xu et al. [26] identified 12 monosaccharides in the EPS synthesized by *P. tenue*, with glucose, galactose, and xylose present at the highest concentrations, and fructose, galactosamine, and fucose found only in traces. Analyzing monosaccharide composition in induced biocrusts of different ages, Colica et al. [40] reported that glucose and galactose were the most abundant monosaccharides, showing the crusts also a high percentage of uronic acids which ranged from 9 to 11 mol%. Two recent studies showed that galactose and glucose were the most abundant components in sandy soils inoculated with *Schizothrix* cf. *delicatissima* and *Leptolyngbya ohadii* [34, 38]. In the present study, up to 12 monosaccharides were found in the different inoculated soil types. The abundance of uronic acids was low, and ribose and fructose were found only in traces (Fig. 6). It is worth mentioning that in the more C-rich soils, i.e., the sandy loam and loamy sand soils, low amounts of the different monosaccharides were also found in the non-inoculated soils. This suggests a possible previous colonization by cyanobacteria in the natural sites where these soils were collected that contributed to the EPS that we found in those soils (Fig. 3).

The number of monosaccharides was similar in the two EPS fractions (LB and TB) extracted from the induced biocrusts. This retraces the findings by Chen et al. [39], who reported a similar compositional pattern in the two EPS fractions. However, there were differences in the relative abundance of the monosaccharides in the two fractions depending on soil type. Glucose was the predominant monosaccharide in both EPS fractions. This sugar represented 47% of LB-EPSs and 87% of TB-EPSs in the sandy soil, but a lower percentage in the other soil types, where it accounted for 44% and 30% in silt loam, 39% and 20% in loamy sand, and 27% and 25% in sandy loam soils, in LB-EPSs and TB-EPSs, respectively (Fig. 6). With the exception of the sandy soil, the resting inoculated soil types also showed high proportions of galactose, mannose, and xylose. The abundance of xylose was particularly high in the TB-EPS fraction from loamy sandy soils (Fig. 6b). Inoculation of the two cyanobacterial strains led to differences in the relative abundance of the monosaccharides in the less C-rich soils. In silty soils, *P. ambiguum* led to higher galactose and lower glucose abundance than *S. javanicum*, while an opposite trend was found in sandy soils (Table S1)*.* In addition, in the sandy soil, *S. javanicum* led to a higher number of monosaccharides and the TB-EPS fraction showed higher fructose and rhamnose abundances (Table S2). The presence of deoxysugars such as fucose and rhamnose confers hydrophobic properties [17, 51] and can increase soil hydrophobicity in cyanobacterial crusts. Despite higher amount of rhamnose was found in the sandy soil inoculated with *S. javanicum* (14%), none of the cyanobacteria-inoculated soils in this study showed hydrophobic properties [23].

### Implications of the Characteristics of the Cyanobacterial Polysaccharidic Matrix for the Environment

This study shows for the first time that the polysaccharidic matrix in induced cyanobacterial crusts shows contrasting macromolecular and chemical features depending on the inoculated strain as well as on soil type. These contrasting features of the cyanobacterial polysaccharidic matrix are likely to condition the abundance, diversity, and activity of microbial communities in desert crusts, as different microorganisms could have different affinities for the use of one or other sugars as source of energy [55, 56]. The high abundance of glucose in sandy soils after cyanobacteria addition can represent the main C source for activity of the heterotrophic community in these very C-poor soils. In soils with more complex chemical properties, the relative abundance of other monosaccharides such as galactose, mannose, and xylose may favor the presence of a more complex and diverse microbial community able to use different sugars as source of energy for their nutrition. The development of this complex polymeric matrix in cyanobacterial biocrusts depending on cyanobacteria species and soil type influences top soil properties such as structure and stability, hydrophobicity and water retention, carbon and nutrient contents and enzymatic activities [3, 15, 19], all of them conditioning successional dynamics of biocrust communities. For instance, large amounts of EPSs excreted by cyanobacteria provide them with a higher metabolic capacity compared to more-developed lichen biocrusts [48]. Mineralization of between 7 and 12% of the total OC has been found in 10 days in cyanobacterial crusts, highlighting the crucial role of cyanobacteria as primary colonizers of bare soils contributing with enzymes that enable degradation of organic substances and favoring the establishment of more developed biocrusts [57]. Stimulation of microbial activity by enhancement in EPS content results in an increase in soil aggregation [21], which favors infiltration and water retention [18, 58]. Greater water availability enhances biological activity of crust organisms, which in turn increases crust biomass, and C and N fixation [59] and thus, the synthesis of more EPSs. This improvement in soil functions suggests that inoculation of EPS-producing cyanobacteria can be considered a promising sustainable technology to foster soil recovery in disturbed dryland soils.

## Conclusions

Cyanobacteria inoculation increased EPS content of soil, with different effects in the amount of the two EPS fractions, LB-EPS and TB-EPS, depending on the cyanobacterial strain inoculated. Inoculation of *S. javanicum* led to a higher release in the soil of the less condensed and more soluble LB-EPSs, which showed a high percentage of low MW molecules and a higher glucose relative abundance, hence likely having relevance as C source readily available for microbial activity. Inoculation of *P. ambiguum* promoted a higher increase of the more condensed TB-EPSs, which were mainly composed of high MW molecules, likely playing an important role in the aggregation of soil particles and improvement of soil structure and stability. Cyanobacteria inoculation differently affected the relative abundance of the monosaccharides in the polysaccharidic matrix of the biocrusts, depending on soil type. In C-poor sandy soils, cyanobacteria inoculation led to the formation of an EPS matrix where glucose was the most abundant monosaccharide. In more C-rich soils, the induced cyanobacterial EPS matrix was mainly composed of glucose, galactose, mannose, and xylose. The presence of fucose and rhamnose that confer hydrophobic properties to EPSs was low, explaining the observed absence of hydrophobicity in the soils inoculated with the selected strains. The different macromolecular and chemical characteristics of the polymeric matrix induced by cyanobacteria in different soil types is likely to modulate changes in soil properties, nutrient cycling, and biocrust successional dynamics, eventually conditioning soil microbial community composition and activity and plant performance in dryland regions.

## Electronic Supplementary Material

ESM 1 (DOCX 15 kb)

ESM 2 (DOCX 15 kb)
