# Supplementary material for: Soil Type and Cyanobacteria Species Influence the Macromolecular and Chemical Characteristics of the Polysaccharidic Matrix in Induced Biocrusts
Source: Microb Ecol. 2018 Dec 8;78(2):482–93. doi: 10.1007/s00248-018-1305-y (PMC6647080; doi:10.1007/s00248-018-1305-y)
Supplement: Supplementary file 1 — (DOCX 15 kb) [file 248_2018_1305_MOESM1_ESM.docx]

Table S1. Monosaccharide composition of the LB-EPS fraction (%, moles of the single monosaccharide/total amount of moles of monosaccharides*100) in the four inoculated soil types. The two-way ANOVA showed a significant (p<0.05) interaction of soil type and inoculum treatment for fu, rha, galN, ara, gal, glc and xyl. The soil type had also a significant effect on glcN and man. No significant effect of soil type or inoculum was found for glcA. Numbers in bold indicate significant differences between the two strains on each soil type. tr is shown when content was lower than 1% and nd is shown for the monosaccharides not detected by the IEC analysis.

|  | Silt loam | | Sandy loam | | Loamy sand | | Sandy | |
| --- | --- | --- | --- | --- | --- | --- | --- | --- |
|  | *P. ambiguum* | *S. javanicum* | *P. ambiguum* | *S. javanicum* | *P. ambiguum* | *S. javanicum* | *P. ambiguum* | *S. javanicum* |
| fuc | **3.31**  (0.89) | **1.35**  (0.20) | 2.78  (0.23) | 2.76  (0.34) | 2.04  (0.44) | 1.67  (0.26) | tr | 1.35  (0.42) |
| rha | 7.61  (0.48) | 4.77  (1.73) | 6.16  (0.53) | 7.87  (0.45) | 7.36  (1.39) | 7.10  (3.93) | tr | 4.67  (1.74) |
| galN | **3.36**  (0.58) | **1.94**  (0.24) | 3.24  (0.07) | 3.61  (0.40) | **3.71**  (0.79) | **2.65**  (0.72) | tr | 1.13  (0.58) |
| ara | **4.87**  (0.95) | **3.05**  (0.44) | 4.72  (0.23) | 4.54  (0.56) | **4.93**  (1.39) | **3.39**  (0.48) | tr | tr |
| glcN | 2.43  (0.27) | 1.90  (0.26) | 3.42  (0.37) | 4.48  (0.43) | 4.05  (1.26) | 3.38  (0.97) | tr | 1.73  (1.12) |
| gal | **21.53**  (3.14) | **13.00**  (1.41) | 17.41  (0.63) | 18.70  (0.81) | 17.36  (0.58) | 13.19  (3.57) | **1.44**  (0.04) | **13.24**  (7.09) |
| glc | **28.45**  (2.84) | **54.55**  (7.27) | 26.92  (1.43) | 26.03  (4.29) | **31.78**  (8.12) | **46.04**  (16.62) | **89.36**  (1.15) | **63.92**  (10.93) |
| man | 11.43  (0.65) | 12.10  (1.38) | 14.45  (1.23) | 15.47  (0.23) | 12.84  (1.67) | 11.60  (2.15) | 5.03  (0.74) | 7.03  (1.10) |
| xyl | 7.19  (0.07) | 6.80  (1.29) | **17.83**  (1.75) | **13.76**  (0.91) | **12.19**  (2.76) | **7.06**  (0.81) | 1.96  (0.21) | 3.25  (0.29) |
| fru | 3.71  (5.25) | nd | nd | nd | tr | nd | nd | nd |
| rib | nd | nd | nd | nd | tr | nd | nd | tr |
| galA | 3.94  (5.57) | nd | tr | tr | tr | tr | nd | tr |
| glcA | 2.17  (3.07) | tr | 2.14  (0.32) | 2.20  (0.18) | 2.28  (0.58) | 3.34  (4.84) | tr | 1.06  (0.66) |

fuc fucose, rha rhamnose, galN galactosamine, ara arabinose, glcN glucosamine, gal galactose, glc glucose, man mannose, xyl xylose, fru fructose, rib ribose, galA galacturonic acid, glcA glucuronic acid.
