# Supplementary material for: Soil Type and Cyanobacteria Species Influence the Macromolecular and Chemical Characteristics of the Polysaccharidic Matrix in Induced Biocrusts
Source: Microb Ecol. 2018 Dec 8;78(2):482–93. doi: 10.1007/s00248-018-1305-y (PMC6647080; doi:10.1007/s00248-018-1305-y)
Supplement: Supplementary file 2 — (DOCX 15 kb) [file 248_2018_1305_MOESM2_ESM.docx]

Table S2. Monosaccharide composition of the TB-EPS fraction (%, moles of the single monosaccharide/total amount of moles of monosaccharides*100) in the four inoculated soil types. The two-way ANOVA showed a significant (p<0.05) interaction of soil type and inoculum type for fu, rha, galN, ara, glcN, man, xyl and fru. The abundance of galactose was significantly affected by inoculum type but not by soil type, while glucose and uronic acids were only significantly affected by soil type. Numbers in bold indicate significant differences between the two strains on each soil type. tr is shown when content was lower than 1% and nd is shown for the monosaccharides not detected by the IEC analysis.

|  | Silt loam | | Sandy loam | | Loamy sand | | Sandy | |
| --- | --- | --- | --- | --- | --- | --- | --- | --- |
|  | *P. ambiguum* | *S. javanicum* | *P. ambiguum* | *S. javanicum* | *P. ambiguum* | *S. javanicum* | *P. ambiguum* | *S. javanicum* |
| fuc | **2.77**  (0.04) | **2.27**  (0.24) | 1.99  (0.03) | 2.00  (0.17) | 1.66  (0.08) | 1.67  (006) | tr | **1.31**  (0.27) |
| rha | 6.02  (0.35) | 7.06  (0.25) | 5.32  (0.45) | 6.24  (1.49) | 3.93  (0.14) | 4.02  (0.24) | **5.05**  (2.70) | **14.09**  (3.07) |
| galN | 1.30  (0.09) | 1.41  (0.23) | 2.93  (0.19) | 3.19  (0.86) | 1.60  (0.08) | 1.37  (0.03) | 1.34  (0.59) | nd |
| ara | **4.24**  (0.39) | **3.36**  (0.48) | 3.21  (0.14) | 3.34  (0.36) | 3.32  (0.11) | 3.19  (0.11) | 1.61  (0.10) | 1.40  (0.20) |
| glcN | 1.53  (0.34) | 1.14  (0.37) | 1.78  (0.06) | 1.92  (0.39) | 1.42  (0.25) | 1.19  (0.08) | tr | nd |
| gal | 22.77  (0.24) | 17.92  (2.75) | 22.13  (0.36) | 23.52  (3.43) | 21.49  (1.84) | 19.64  (0.59) | **21.09**  (5.68) | **14.55**  (4.52) |
| glc | 26.90  (0.94) | 34.04  (1.64) | 27.02  (0.61) | 22.56  (10.11) | 20.12  (1.84) | 20.48  (2.37) | 49.90  (6.26) | 43.77  (2.96) |
| man | **12.57**  (0.28) | **9.67**  (0.38) | **15.77**  (0.22) | **13.57**  (0.51) | 14.73  (0.50) | 14.17  (0.63) | **9.16**  (0.80) | **5.69**  (0.86) |
| xyl | **12.13**  (0.77) | **9.78**  (1.00) | 10.47  (0.81) | 11.43  (0.69) | 22.87  (1.22) | 23.28  (0.78) | 5.38  (0.35) | 4.53  (0.93) |
| fru | nd | **4.59**  (2.51) | tr | tr | tr | tr | **1.89**  (1.94) | **11.17**  (1.80) |
| rib | nd | tr | nd | nd | nd | nd | nd | 2.31  (0.63) |
| galA | 4.11  (0.22) | 3.40  (0.22) | 4.54  (0.62) | 6.41  (2.18) | 3.96  (3.51) | 6.28  (0.54) | nd | nd |
| glcA | 5.65  (0.58) | 4.56  (0.29) | 4.24  (0.31) | 5.42  (1.79) | 4.39  (0.44) | 3.79  (0.31) | 3.10  (2.70) | 1.17  (2.02) |

fuc fucose, rha rhamnose, galN galactosamine, ara arabinose, glcN glucosamine, gal galactose, glc glucose, man mannose, xyl xylose, fru fructose, rib ribose, galA galacturonic acid, glcA glucuronic acid.
